# Development of a measure of model fidelity for mental health Crisis Resolution Teams

**DOI:** 10.1186/s12888-016-1139-4

**Published:** 2016-12-01

**Authors:** Brynmor Lloyd-Evans, Gary R. Bond, Torleif Ruud, Ada Ivanecka, Richard Gray, David Osborn, Fiona Nolan, Claire Henderson, Oliver Mason, Nicky Goater, Kathleen Kelly, Gareth Ambler, Nicola Morant, Steve Onyett, Danielle Lamb, Sarah Fahmy, Ellie Brown, Beth Paterson, Angela Sweeney, David Hindle, Kate Fullarton, Johanna Frerichs, Sonia Johnson

**Affiliations:** 1 Division of Psychiatry, UCL, 149 Tottenham Court Road, London, W1T 7NF UK; 2 Department of Psychiatry, Dartmouth Psychiatric Research Center, Geisel School of Medicine at Dartmouth, Lebanon, NH 03766 USA; 3 Division Mental Health Services, Akershus Unieversity Hospital, Lørenskog, Norway; 4 Institute of Clinical Medicine, University of Oslo, Oslo, Norway; 5 Mental Health Sciences Department, University of the West of England, Coldharbour Lane, Bristol, BS16 1QY UK; 6 Research Department of Clinical, Education and Health Psychology, University College London Gower Street, London, WC1E 6BT UK; 7 Institute of Psychiatry, Psychology and Neuroscience, Kings College London, 16 De Crespigny Park, London, SE5 8AF UK; 8 West London Mental Health NHS Trust, Uxbridge Road, Southall, London, UB1 3EU UK; 9 Oxfordshire Healthcare NHS Foundation Trust, Barnes Unit, John Radcliffe Hospital, Oxford, OX3 9DU UK; 10 Department of Statistical Science, UCL, Gower Street, London, WC1E 6BT UK; 11 Onyett Entero Ltd, care of University of the West of England, Health and Life Sciences Coldharbour Ln, Bristol, BS16 1QY UK; 12 School of Psychology, University of Surrey, Guildford, Surrey GU2 7XH UK

**Keywords:** Crisis Resolution Team, Mental health services, Acute care, Fidelity, Implementation

## Abstract

**Background:**

Crisis Resolution Teams (CRTs) provide short-term intensive home treatment to people experiencing mental health crisis. Trial evidence suggests CRTs can be effective at reducing hospital admissions and increasing satisfaction with acute care. When scaled up to national level however, CRT implementation and outcomes have been variable. We aimed to develop and test a fidelity scale to assess adherence to a model of best practice for CRTs, based on best available evidence.

**Methods:**

A concept mapping process was used to develop a CRT fidelity scale. Participants (*n* = 68) from a range of stakeholder groups prioritised and grouped statements (*n* = 72) about important components of the CRT model, generated from a literature review, national survey and qualitative interviews. These data were analysed using Ariadne software and the resultant cluster solution informed item selection for a CRT fidelity scale. Operational criteria and scoring anchor points were developed for each item. The CORE CRT fidelity scale was then piloted in 75 CRTs in the UK to assess the range of scores achieved and feasibility for use in a 1-day fidelity review process. Trained reviewers (*n* = 16) rated CRT service fidelity in a vignette exercise to test the scale’s inter-rater reliability.

**Results:**

There were high levels of agreement within and between stakeholder groups regarding the most important components of the CRT model. A 39-item measure of CRT model fidelity was developed. Piloting indicated that the scale was feasible for use to assess CRT model fidelity and had good face validity. The wide range of item scores and total scores across CRT services in the pilot demonstrate the measure can distinguish lower and higher fidelity services. Moderately good inter-rater reliability was found, with an estimated correlation between individual ratings of 0.65 (95% CI: 0.54 to 0.76).

**Conclusions:**

The CORE CRT Fidelity Scale has been developed through a rigorous and systematic process. Promising initial testing indicates its value in assessing adherence to a model of CRT best practice and to support service improvement monitoring and planning. Further research is required to establish its psychometric properties and international applicability.

**Electronic supplementary material:**

The online version of this article (doi:10.1186/s12888-016-1139-4) contains supplementary material, which is available to authorized users.

## Background

Fidelity measures are tools to assess the implementation of intervention or programme models [1], and as such, can help address the major challenge for mental health services of translating scientific knowledge into patient benefit [2]. Development of fidelity measures for complex interventions in mental health services has been advocated not only as a means to define an intervention and measure services’ adherence to the model specified, but also to support service improvement [1]. The US Evidence-Based Practice Program demonstrated that a service improvement initiative involving fidelity measurement as a key component led to successful implementation of five different evidence-based practices in a majority of services, in a large-scale, national programme [3]. Fidelity scales become credible measures of service quality when higher fidelity scores have been shown to be associated with better services outcomes, as for instance with a fidelity scale measuring evidence-based supported employment [4]. Fidelity scales have been developed for complex mental health services, such as Assertive Community Treatment [5] but there is no existing fidelity scale for Crisis Resolution Teams.

Crisis Resolution Teams (CRTs) provide short-term, intensive home treatment to people experiencing a mental health crisis, with the aim of averting hospital admission wherever possible, or supporting people to return home as promptly as possible following an acute admission [6]. The CRT model has not been highly specified in the literature, leading to diverse approaches to implementing these services. Key characteristics of CRTs recommended in government and expert guidance are that: CRTs should provide an easy access, rapid response, 24 h service; should be multi-disciplinary and able to provide medical, psychological and social interventions; should help facilitate prompt discharge from acute wards; and should fulfil a “gatekeeping” function of assessing all patients before admission to acute wards and considering home treatment as an alternative to admission wherever possible [7, 8]. CRTs have been implemented on the largest scale in the UK, where they were mandated in England by the NHS Plan in 2000 [9]. They also form part of national mental health policy in Norway [10] and have been implemented regionally in a number of countries including Australia and the Netherlands.

CRTs are one form of home-based crisis intervention. A recent systematic review from the Cochrane Collaboration concluded that home-based crisis intervention can be an effective alternative to hospital admission [11]. This review included only one randomised trial of a UK CRT service [12], which found that a CRT reduced hospital admissions and inpatient bed use, and increased service users’ satisfaction with acute care. Similarly positive results have been found in non-randomised studies [13, 14]. However, the potential benefits of CRT services suggested in research studies have not been fully translated into practice. Two analyses from a UK nationwide study using routine hospital admissions data reached conflicting conclusions about whether there is any association between the introduction of CRTs to a local area and a reduction in bed use [15, 16]. Rates of compulsory inpatient admissions in the UK have risen over the last decade despite CRT implementation [17, 18]. Dissatisfaction from service users with CRT care has also been reported in recent national reports [19, 20]. These findings may reflect the incomplete and inconsistent CRT implementation in the UK: a national survey by Onyett and colleagues [21] found that only 40% of CRTs considered themselves fully implemented as intended, with wide variation in teams’ organisation and service delivery. Similar variation in CRT services’ characteristics was found in a more recent UK survey [22]. In Norway too, emerging evidence suggests that CRTs are providing a less intensive, less frequently home-based service, to a less acutely unwell client group than originally intended, with a consequent diminished impact on averting hospital admissions [10]. A recent systematic review [23] found little empirical evidence about the critical ingredients of CRTs, but found that there are indications from qualitative research, surveys and guidelines about which aspects of CRT service delivery and organisation are considered important or helpful by stakeholders.

The lack of a clearly specified CRT model, and the suggestion that potential benefits of CRTs are not being consistently achieved when services are scaled up to a national level, indicate the need for a rigorously defined and well-validated CRT fidelity scale. In the absence of a clearly prescribed theoretical model or sufficient empirical evidence about the critical ingredients of an intervention or service model, stakeholders’ views regarding best practice may also inform the development of fidelity criteria. Structured approaches used in fidelity scale development to elicit stakeholder opinion have included a Delphi process [24] and concept mapping [25]. Stakeholder groups often include researchers, program leaders, practitioners, and service users [26, 27]. In this study, we aimed to systematically develop a fidelity scale for CRTs; to test the feasibility and utility of the scale in practice settings; and to conduct a preliminary exploration of its psychometric properties. This work was undertaken as part of a larger research programme on implementation of CRTs, the CORE Study [28]. The CORE Study programme as a whole aimed to develop evidence to inform effective CRT implementation. It involved: i) developing evidence regarding the optimal CRT model from a systematic literature review, a national survey, and interviews with a range of stakeholders; ii) development and testing of a measure to assess model fidelity in CRTs (the work reported in this paper); and iii) development and testing in a cluster randomised trial of a package of service improvement resources designed to enhance model fidelity and improve outcomes in CRT teams. Further information about the CORE Study as a whole is available from the study website [29] and the trial protocol for the service improvement programme trial [30].

## Methods

Development of the CRT fidelity scale consisted of three steps: construction of the scale; piloting and refinement; and exploration of its psychometric properties.

### Construction of the fidelity scale

Concept mapping was proposed by [31] as a structured process to facilitate group participation in developing conceptual frameworks to guide evaluation. We used a concept mapping process to construct the CRT fidelity scale, following the six stages described by Trochim of: i) developing the focus for conceptualisation; ii) generating statements; iii) group participation in conceptualising (grouping) and prioritising statements; iv) representing these statements in a concept map; v) interpreting the map; vi) utilising the map.

i. Developing the focus for conceptualisation: Potential characteristics of CRT resources, organisation and service delivery for inclusion in a fidelity scale were identified from three sources: a literature review of quantitative and qualitative studies and guidelines relating to CRT implementation [23]; a UK national survey of CRT managers, reporting description of teams’ organisation and service delivery and managers’ views on priorities for effective CRT implementation [22]; and over 100 interviews and focus groups with CRT stakeholders (CRT service users, carers, staff and managers; and other stakeholders from organisations which refer to or work with CRTs) conducted for the CORE study [28]. The list of potential CRT fidelity characteristics was also confirmed by the results from a similar survey of the 56 CRT managers in Norway and a qualitative study of experiences of service users, carers, team members and collaborating services in Norway [32].
ii. Generating statements: From a “longlist” of potential components of a CRT model generated in stage 1, a group of CRT stakeholders (*n* = 10), comprising clinicians and academic researchers, including service user-researchers, were asked to develop a set of fewer than 100 statements specifying potential components of a CRT fidelity scale. Participants completed this task as a group exercise, through discussion and manual sorting of cards (each with a statement on it). At this stage, participants were asked to de-duplicate, collapse or combine conceptually related statements rather than to judge the relative importance of statements or exclude any distinct areas of CRT organisation or practice included in the statements.
iii. Group participation in conceptualising statements: Stakeholder involvement was sought through concept mapping meetings. Participants were invited to one of four meetings, held in London, UK (*n* = 2), Northampton, UK (*n* = 1) or Oslo, Norway (*n* = 1). Participants who could not attend a meeting could complete the concept mapping tasks individually and return their results to the research team. Participants were sought from the following six CRT stakeholder groups: i) service users; ii) family and friends supporting service users (carers); iii) CRT staff; iv) other mental health staff (including senior managers and staff from mental health services which work with CRTs, including acute wards and Community Mental Health Teams; v) staff from voluntary sector organisations providing support to people with mental health problems; and vi) academic researchers involved in acute care research. Participants were convenience sampled from service user and carer research groups, clinical professional networks and a clinicians’ advisory group already assembled for the CORE Study, UK and Norwegian clinical and research networks, and via a large national mental health charity in England (MIND).
  Participants were given two sets of cards, each set with a set of statements relating to service organisation or delivery in CRTs. Participants were also provided with an accompanying sheet with brief information clarifying the meaning of each statement and presenting a rationale for its inclusion (based on development work from stage 1, and indicating whether any empirical evidence, policy guidance or evidence of stakeholders’ views supported its inclusion). First, participants were asked to group cards together into a minimum of two groups, according to the participant’s view of how the statements best fit together, and to name each group (the conceptualisation task). Secondly, participants were asked to sort the cards into five equal-sized groups, identifying those viewed as most important, next most important, down to least important for delivering an effective CRT service (the prioritisation task). Participants completed the tasks individually, without discussion. Research staff were present to help explain the tasks where necessary and record results from each participant.
iv. Representation of statements in a concept map: Participants’ data from the concept mapping exercises were entered into a specialist concept mapping software programme “Ariadne” [33]. Ariadne generates outputs regarding the mean importance ratings for each statement (for all participants and participant groups, with each statement scoring on a scale 1–5 for each participant, based on their prioritisation of items). Using principal component analysis and cluster analysis, it generates a series of concept maps, identifying how statements can best be grouped together in cluster solutions ranging from 2–20 clusters. The concept maps are shaped based on the participants’ conceptualisation of statements.
v. Interpretation of concept maps: A stakeholder group (*n* = 8) including CRT service users, carers, mental health staff and academics reviewed the 19 cluster solutions generated by Ariadne. With reference to the statements grouped within each cluster for each solution, group members were asked to: a) Select the cluster solution with greatest conceptual coherence; and b) name each cluster (referring back to participants’ naming of groups in the concept mapping exercise). A consensus was sought through discussion.
vi. Utilising the concept map: The chosen cluster solution, i.e. the final concept map, was then used as a basis for developing the CRT fidelity measure. Statements representing each concept map cluster (i.e. each CRT conceptual domain) were included in the fidelity scale. Decisions regarding the number of statements from each cluster to be included in the scale were guided by the mean importance score overall for statements within each cluster, as well as the number of statements the cluster contained. Statements with higher mean importance scores within each cluster were prioritised for inclusion. The mean importance scores from each participant group were also inspected for each cluster and (with participants regrouped into three broader groups – service users and carers, mental health staff, others), compared using bivariate tests for individual items: additional consideration was given to items prioritised by any respondent group.
  Once statements for inclusion in a fidelity measure had been selected, the research team transformed these essential components into items useful for a CRT fidelity scale, by developing operational definitions and scoring anchor points for each item. This was achieved through an iterative process of reviews of evidence on CRT functioning and, where possible, evidence which supported the dosing of interventions. The resultant fidelity items allowed adherence to each item to be scored on a five-point scale, in keeping with other well-established fidelity measures [34, 35]. At each stage, further stakeholder consultation was sought from: concept mapping participants, advisory working groups of service user and carer researchers attached to the CORE Study, clinical networks (e.g. the Royal College of Psychiatrists CRT network) and available CRT experts. Views were sought regarding: whether the criteria are valid – i.e. do they belong in a model of best practice for CRTs, whether the criteria are attainable in routine service settings; and whether criteria could be reliably measured during an audit process.

### Piloting

To pilot the fidelity scale and test its feasibility as a measure of model adherence for CRTs, a 1-day fidelity review process was developed. This followed the processes for assessing fidelity developed for established scales [34, 35]. Reviews were carried out by a team of three reviewers, who visited a CRT service for a full day. To prepare for the fidelity review, a member of the review team contacted the CRT management prior to the visit, explaining the purpose, identifying documents the review team would need, and setting up the schedule. Review teams included at least one mental health clinician and one service user or carer, as well as one member of the study research team. Reviews involved: interviews with multiple groups (the CRT manager and staff team, managers of other services which work closely with the CRT, 6 CRT service users and 6 carers); a review of anonymized case records for the 10 most recent, consecutively discharged, service users; and review of service policies, records and routinely collected data. Reviewers collected evidence using during the review day using interview schedules and checklists provided to them, then met to share information and collectively score each fidelity item at the end of the review day. A draft fidelity review report was then sent to the CRT manager, seeking clarification on any outstanding issues and offering an opportunity for the CRT manager to query any scores and provide further evidence if available, before a finalised report and score were provided. A half-day training programme and electronic training materials were provided in advance to all reviewers, in which interview schedules, checklists and scoring guidance for use during reviews were provided.

Piloting of the fidelity scale and review process was planned in two stages; an initial pilot of four teams, then a larger programme of reviews in 75 teams. The protocol for this programme of reviews was confirmed by the London Camden and Islington Research Ethics Committee as meeting criteria for audit rather than research [36], so approvals from participating NHS organisations only were required. Study researchers contacted CRT managers about willingness to participate in a fidelity review. Where the CRT manager was interested, the NHS Trust Research and Development Department was contacted: any registration or application processes required by Trusts to conduct the fidelity review as an audit were complied with. For each review, the manager of the participating CRT service invited other staff, service users and carers to take part in the review and the review team provided an information sheet for potential service user and carer participants. The review team also confirmed the team’s willingness to take part in the review, and provided all participants an opportunity to ask questions on the review day.

The review team sought feedback from the manager of all participating CRT teams regarding the acceptability of the review process, clarity and validity of the fidelity scale criteria, and whether scoring anchor points for individual items were set appropriately. The research team monitored the range of scores being achieved for each item, to identify potential floor or ceiling effects in scale items. A review of the scale was planned after 50 reviews, to allow use of later reviews to test a Version 2 of the scale if necessary.

### Psychometric properties

Three properties of the CRT fidelity scale were explored. Feedback from managers of CRT services undergoing a fidelity review was used to assess the scale’s *face validity*. The range of scores generated in the 75-team survey, for total and item scores, was used to *assess for restricted range* (e.g., floor or ceiling effects). Reviewers’ *inter-rater reliability* was tested using an extended vignette. Researchers (JF, KF, DL) developed mock interview transcripts and audit records for a fidelity review, informed by records from actual reviews. Reviewers from the 75-team survey were then invited to score each of the scale’s 39 fidelity items with reference to the mock records, as an individual exercise. Reviewers’ scores for each item were entered into Stata Statistical Software version 13 for analysis. Absolute agreement intra class correlations were calculated, based on a two-way random effects model [37].

## Results

### Construction of the fidelity scale

An initial list of 232 statements relating to recommended characteristics of CRT services was collapsed to 72 statements regarding good practice in CRTs’ organisation and service delivery. In total, there were 68 participants in the concept mapping exercises, including representatives of all six stakeholder groups, and participants from the UK and Norway, the Netherlands and Australia. Participants’ characteristics are summarised in Table 1.

**Table 1:** Characteristics of participants in the concept mapping exercise (*N* = 68)

Participants		Number: *n* (%)
Stakeholder group	CRT staff	22 (32%)
	Service users	16 (24%)
	Academic researchers	10 (15%)
	Other mental health staff	8 (12%)
	Carers	6 (9%)
	3rd sector staff	6 (9%)
	Total	68 (100%)
Country of CRT contact	UK	56 (82%)
	Norway	10 (15%)
	Australia	1 (1%)
	Netherlands	1 (1%)
Gender	Male	25 (37%)
	Female	42 (63%)
	Not reported	1
Ethnicity	White British	39 (58%)
	White Irish	3 (4%)
	White other	18 (27%)
	Black African/Caribbean	2 (3%)
	Asian	1 (1%)
	Mixed or other ethnic groups	4 (6%)
	Not reported	1

**Fig. 1:**
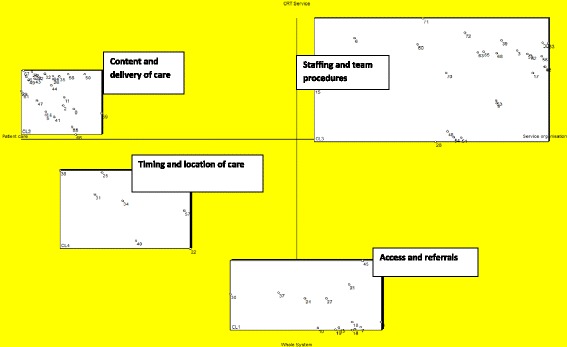
CORE CRT concept map: the chosen 4-cluster solution

Participants grouped statements into between 2–12 groups. Mean and median importance scores for each statement are provided in the data supplement (Additional file 1: Table DS1). A four-cluster solution was selected as the most conceptually coherent model (Fig. 1). The stakeholder working group (*n* = 8) named the clusters as: *referrals and access; content and delivery of care; staffing and team procedures; timing and location of care*.

The three broadly defined stakeholder groups (service users and carers; mental health staff; others) showed high agreement in the importance ratings of most items. Bivariate tests found significant differences between stakeholder groups in mean importance ratings for 15 (21%) of 72 statements. All statements rated very important (mean score above 4) by any of the three groups, or moderately important (mean score above 2.5) by more than one stakeholder group were included in the scale, either as a distinct item, or among item scoring criteria. Additional file 2: Table DS2 provides further information and an explanation of how discrepancies between stakeholder groups’ ratings were accounted for in development of the fidelity scale.

Thirty nine statements were selected for inclusion in the CRT Fidelity Scale. Table 2 reports how the internal structure of the fidelity scale relates to the clusters and their mean importance rating from the concept map. Statements representing each of the four clusters were included in the fidelity scale: the relative contribution to the total scale and score of each cluster was guided by the number of statements, and the mean importance rating for statements, within each cluster. Further consultation (within the study team, with study working groups of service users and carers, with concept mapping participants, and with other available CRT experts) following the concept mapping process, resulted in slightly greater representation in the scale of items relating to the content of care than was indicated by the concept map.

**Table 2:** The relationship of the CRT fidelity scale structure to concept map clusters

Cluster	Number of statements from concept mapping (*N* = 72)	Mean of mean importance scores for statements in this cluster	Number of statements in the CRT fidelity scale (*N* = 39)
1. Referrals and access	14 (19%)	3.41	10 (26%)
2. Content and delivery of care	26 (36%)	2.84	16 (41%)
3. Staffing and team organisation	25 (35%)	2.98	10 (26%)
4. Location and timing of care	7 (10%)	3.15	3 (8%)

#### Piloting

During initial piloting in four CRT services we refined scoring criteria and guidance to make the wording clearer. This resultant revised version of the fidelity scale (“Version 1”) was then piloted in 75 CRTs in England, Scotland and Wales, serving a range of inner city, suburban and more rural areas, and catchment area populations ranging from about 100,000 to 400,000. The 75 CRTs surveyed included 70 in England – about a third of CRT teams within England. Reviews were completed successfully and a report and score generated for all 75 teams which agreed to take part in the survey. Feedback from CRT managers was that the fidelity reviews were acceptable and the reports helpful, but that the preparation required by the team was time-consuming and at the upper limit of what was manageable. Fidelity reviews required long days (typically 8–10 h), with scoring on occasion being completed by reviewers after the scheduled finish time.

The information required by reviewers was not always all available on the review day: in particular, six service users and carers were not always contactable or available. Wherever possible, reviewers sought to obtain missing information later (for instance by returning to a service for a second day, or conducting interviews by phone). Services were only scored on the information provided: some teams may therefore have scored lower than they might have. CRT managers also reported that for some items, low scores may have reflected poor documentation in case notes of care provided, rather than a lack of care itself.

This 75-team survey yielded a range of total fidelity scores of 73–151 (maximum possible range 39 – 195). At item level, the maximum range of scores (1–5) was obtained for 33 items; 4 items (items 2, 4, 15 and 39) yielded a range of scores from 2–5; and 2 items (items 16 and 24) yielded a range of scores from 1–4. Thirty one items yielded a median score in the range 2–4. For three items, a majority of teams received the minimum score of 1 (item 17 (51% scored 1), item 24 (80%) and item 37 (56%)). For five items, a majority of teams received the maximum score of 5 (items 19 (51% scored 5), 26 (53%), 27 (63%), 32 (56%) and 39 (56%)). The median score and range of scores for each item are reported in Table 3.

**Table 3:** CORE CRT Fidelity Scale - item median scores and range of scores from pilot (*n* = 75)

	#	Item	Median score	Range
Referrals and Access	1	The CRT responds quickly to new referrals	2	1–5
	2	The CRT is easily accessible to all eligible referrers	4	**2–5**
	3	The CRT accepts referrals from all sources	3	1–5
	4	The CRT will consider working with anyone who would otherwise be admitted to adult acute psychiatric hospital	4	**2–5**
	5	The CRT provides a 24 h, 7 day a week service	4	1–5
	6	The CRT has a fully implemented “gatekeeping” role, assessing all patients before admission to acute psychiatric wards and deciding whether they are suitable for home treatment.	4	1–5
	7	The CRT facilitates early discharge from hospital	3	1–5
	8	The CRT provides explanation and direction to other services for service users, carers and referrers regarding referrals which are not accepted	4	1–5
	9	The CRT responds to requests for help from service users and carers whom the CRT is currently supporting	3	1–5
	10	The CRT is a distinct service which only provides crisis assessment and brief home treatment	4	1–5
Content and delivery of Care	11	The CRT assertively engages and comprehensively assesses all service users accepted for CRT support	4	1–5
	12	The CRT provides clear information to service users and families about treatment plans and visits	3	1–5
	13	The CRT closely involves and works with families and wider social networks in supporting service users	3	1–5
	14	The CRT assesses carers’ needs and offers carers emotional and practical support	2	1–5
	15	The CRT reviews, prescribes and delivers medication for all service users when needed	5	**2–5**
	16	The CRT promotes service users’ and carers’ understanding of illness and medication and addresses concerns about medication	2	**1–4**
	17	The CRT provides psychological interventions	1	1–5
	18	The CRT considers and addresses service users’ physical health needs	2	1–5
	19	The CRT helps service users with social and practical problems	5	1–5
	20	The CRT provides individualised care	3	1–5
	21	CRT staff visits are long enough to discuss service users’ and families’ concerns	3	1–5
	22	The CRT prioritises good therapeutic relationships between staff and service users and carers	2	1–5
	23	The CRT offers service users choice regarding location, timing and types of support	4	1–5
	24	The CRT helps plan service users’ and service responses to future crises	1	**1–4**
	25	The CRT plans aftercare with all service users	3	1–5
	26	The CRT prioritises acceptability to service users in how CRT care is ended	3	1–5
Staffing and team procedures	27	The CRT has adequate staffing levels	5	1–5
	28	The CRT has a psychiatrist or psychiatrists in the CRT team, with adequate staffing levels	5	1–5
	29	The CRT is a full multi–disciplinary staff team	2	1–5
	30	The CRT provides a thorough induction programme for new staff and ongoing training and supervision in core competencies for CRT staff	3	1–5
	31	The CRT has comprehensive risk assessment and risk management procedures, including procedures for safeguarding children and vulnerable adults living with CRT service users	2	1–5
	32	The CRT has systems to ensure the safety of CRT staff members	5	1–5
	33	The CRT has effective record keeping and communication procedures to promote teamwork and information sharing between CRT staff	4	1–5
	34	The CRT works effectively with other community services	3	1–5
	35	The CRT takes account of equality and diversity in all aspects of service provision	4	1–5
	36	The CRT has systems to provide consistency of staff and support to a service user during a period of CRT care	2	1–5
Timing and location of care	37	The CRT can access a range of crisis services to help provide an alternative to hospital admission for service users experiencing mental health crisis	1	1–5
	38	The CRT provides frequent visits to service users	2	1–5
	39	The CRT mostly assesses and supports service users in their home	5	**2–5**

*Range scores of less than 1-5 are presented in bold

The research team reviewed Version 1 of the fidelity scale following 50 reviews. Additional file 3: Table DS3 summarises the changes made to the scale following this review and their rationale. Changes were made to items and scoring criteria in response to: i) feedback from CRT managers and teams during the survey about the validity or items; ii) feedback from reviewers about the clarity of items or how possible it was to retrieve the required information during a review day; iii) floor or ceiling effects observed from the range and variance of scores for individual items which limited their ability to distinguish higher and lower fidelity services; and iv) further consultation with stakeholders, including review of new government and expert guidance for CRTs published during the survey. All 39-items were retained. New criteria were added to four items, and criteria were dropped, amended or combined in an additional 15 items.

The refined, Version 2 of the CRT Fidelity Scale was used alongside Version 1 in the last 9 reviews of the 75-team survey (reviewers scored both versions). Teams’ total scores were a mean of 2 points higher on V2 compared to V1 of the scale (range 0–6 points). For each of the two items with most marked floor effects in the pilot (items 16 and 24), three teams increased their score by one point in V2 compared to V1, and six teams’ scores were unchanged. The effect of the changes to Version 2 of the scale on teams’ overall scores was therefore modest. The feedback from reviewers and participating services was that the changes increased the clarity of the scale and retained good face validity.

#### Psychometric properties

Seventeen reviewers participated in the inter-rater reliability exercise using Version 2 of the CRT fidelity scale and an extended case note vignette. Sixteen participants provided complete data which were used in analysis. These included service user and carer, clinician, and non-clinical researcher reviewers.

The estimated correlation between individual ratings was 0.65 (95 CI: 0.54 to 0.76), indicating reasonably high similarity between ratings within an item. The estimated intra-class correlation (ICC) between ratings averaged over the 16 raters was very high, 0.97 (0.95 to 0.98). Consistency of agreement ICCs produced nearly identical values to those above; the average consistency of agreement ICC (0.97, above) is equivalent to Cronbach’s alpha.

Mean scores and the standard deviation were calculated for each item to establish which items showed the most variability (high SD), and are provided in Additional file 4: Table DS4. It is unclear, however, from this single vignette, whether these results reflect the inherent reliability of scale items, or the clarity of the specific information within this vignette.

## Discussion

### Main findings

This paper describes the development of the first measure of model fidelity for Crisis Resolution Teams: the CORE CRT Fidelity Scale. The CRT model specified within the scale is based on best available evidence and stakeholders’ priorities for CRT service delivery and organisation. The development of the scale has followed a structured and transparent concept mapping process. The 75-team pilot shows that the resulting CRT Fidelity Scale and review process have good feasibility and acceptability for use in CRTs. Results from the pilot show that the scale can distinguish higher and lower fidelity services overall, and that most items also generate a fairly balanced spread of scores across items’ five point scoring range. A minority of items where high scores were obtained by either very few or very many teams were retained with minimal changes for two reasons: first, further consultation with stakeholders confirmed that these items accurately describe important components of the CRT service model; second, a national survey of CRTs undertaken as development work for the fidelity scale [22], and the fact that at least some teams in the 75-team pilot scored good or excellent fidelity, both suggest that the scoring criteria are attainable in practice.

A 9-team pilot of version 2 of the CORE CRT Fidelity Scale suggests it generates only modest changes to total and item scores compared to version 1, but increases the clarity of the scale. The inter-rater reliability testing of V2 of the CORE CRT Fidelity Scale indicates promising initial psychometric properties.

### Limitations

Two limitations of the scale development work reported here are: the scope of consultation in the development of the scale; and the extent of psychometric testing conducted.

First, regarding consultation, we aimed to include all stakeholder groups’ views at all stages of the development of the scale through: i) a thorough review of available evidence, including qualitative research, surveys and guidance as well as empirical studies at the statement-generating stage; ii) inclusion of service user, clinical staff and other stakeholder representatives at statement selection, concept mapping and concept mapping cluster solution-stages; and iii) consultation with service users, carers and clinicians at the fidelity item development stage through research forums and national networks. However, participants in our concept mapping exercise were pragmatically recruited: clinicians slightly outnumbered service users and carers, and some important stakeholder groups (e.g. General Practitioners, service commissioners, emergency services staff (e.g. police and ambulance) were not represented. Most of those who participated in consultations were UK stakeholders: while a small number of participants from the Netherlands, Norway and Australia contributed to the concept mapping exercise, how far the CRT fidelity scale reflects the priorities of stakeholders in other countries, and is suitable for use outside the UK, remains to be investigated.

Second, more extensive testing of the psychometric properties of the CRT Fidelity Scale is desirable: for example, assessing the test-retest reliability of the scale. This was not attempted in our study. The demands and time consumed by a fidelity review day and preparation for it are considerable for participating CRT services. Expecting CRT teams to participate in a second review soon after the first, which would provide no additional benefit to the service, was therefore not feasible.

Exploring inter-rater reliability in vivo was also not attempted. During a fidelity review, the three reviewers all collect evidence from different sources, then meet at the end of the day to share information and agree fidelity scores. Because no single reviewer holds all the required information until scores are discussed, individual reviewers could not assess services’ fidelity independently, to allow comparison of scores.

The vignette exercise used to assess inter-rater reliability drew on information collected from actual reviews (anonymised, and combined from multiple services), giving the exercise a high degree of realism. It only tested the reliability of reviewers’ ratings from the available evidence however, rather than how consistently reviewers gather evidence during a review day. Moreover, conclusions about the inter-rater reliability of the CRT Fidelity Scale, when based on ratings from a single vignette, can only be viewed as provisional. Arguably however, the vignette exercise provided a harder test of inter-rater reliability than an actual review, in that participants in the exercise made their scores entirely independently. In a real fidelity review, by contrast, three reviewers discuss the evidence collected and how to score a service, and refer back to the CRT manager or others to collect more information where necessary. In this light, the moderately good inter-rater reliability of the scale indicated by the vignette exercise, is promising, and suggests the reviewer training and scoring guidance within the scale are adequate to allow the scale to be used reliably.

The rigorous initial evidence gathering, and positive feedback from stakeholders and CRT teams which participated in piloting, demonstrate that the CRT Fidelity Scale has good face validity. The concept mapping grouping exercise, and cluster structure underpinning the scale, also afford it good content validity. However, the criterion validity of the scale, i.e. the relationship between a high fidelity score and outcomes for CRT services, has yet to be explored. This step is critical to establishing the utility of a fidelity scale [38]. Establishing criterion validity for the scale as well as for individual items is of particular importance, given the lack of empirical evidence about critical ingredients of CRT services [23] which was available to inform scale development during the development of statements about CRT best practice or stakeholders’ prioritisation of statements in the concept mapping process.

### Clinical implications

The successfully completed 75-team pilot suggests that the fidelity scale and accompanying audit process, involving clinician and service user or carer-reviewers, is feasible and acceptable to CRT services. Experience from piloting suggests three features of the review process can help to maximise the reliability of fidelity reviews and scoring, and thus enhance the potential clinical utility of the scale. First, reviewing teams should include at least three reviewers, to manage the workload of a review day, provide a range of expertise to inform the evidence review, and to help the reliability of the review scoring process by moderating any outlying views of individual reviewers. Second, using the interview guides and checklists developed for reviewers aids consistent collection and recording of information with which to score a team using the scale. Third, a right of reply by the CRT team manager to an initial draft of the fidelity report and scores can allow any additional evidence to be provided, or misunderstandings by the reviewing team to be corrected.

The CRT Fidelity Scale can be a useful tool for service planners, managers and commissioners in three ways. First, it provides clear specification of the CRT model, in more detail than has been previously provided in statutory or expert guidance [7, 39]. This can guide service planners in setting commissioning specifications for Crisis Resolution Teams, and guide CRT managers and staff in setting service improvement goals. Second, a fidelity review using the CRT Fidelity Scale, which provides services with a score and feedback on each of the scale’s 39 items, can help CRT teams recognise their existing strengths and areas where service development is required. It can thus help teams to make service improvement plans and assess the impact of quality improvement initiatives. We have demonstrated that an external audit is feasible, acceptable, and, preliminary evidence suggests, adequately reliable. The CRT Fidelity Scale could in principle also be used for internal audit within health organisations, or self-audit by teams (although the reliability of scores when the scale is used this way is yet to be tested). Third, at a local or national level, the CRT Fidelity Scale can be used to generate benchmarking data about CRTs’ model fidelity, against which individual teams’ model fidelity can be assessed, or with which changes to service provision across a region or country over time could be explored. Our 75-team survey demonstrates that such a large-scale use of the CRT Fidelity Scale can be achieved. (All 75 reviews in our study were completed in less than 1 year.)

The value of the CRT fidelity scale to service planners and policy-makers is demonstrated by the speed with which it has been promoted and used by expert bodies and policy-making organisations nationally in the UK. The CRT Fidelity Scale is advertised as a “national inspiration” in the Crisis Care Concordat campaign led by NHS England [40]. Benchmarking data from our 75-team survey have been used by the Care Quality Commission, the body responsible for quality inspections of health and social care services in England [20], and the Royal College of Psychiatrists [41] in recent reports presenting recommendations for CRT care.

### Research implications

An important next step in developing the CRT Fidelity Scale is investigating its criterion validity. Fidelity scales assess elements of service or intervention structure and process: these can only be legitimately used as measures of service quality if a positive relationship to outcomes has been demonstrated [42]. Investigating the relationship of teams’ CRT Fidelity Scale score to important outcomes like service users’ experience of and satisfaction with services, recovery following CRT care, and inpatient admission rates and bed use across a catchment area, is of high research interest. An initial exploration of the criterion validity of the CRT Fidelity Scale will be carried out in connection with a current trial of a CRT service improvement programme, also conducted as part of the CORE Study [30].

A further question is whether the CRT Fidelity Scale can be an internationally useful measure, as other Fidelity Scales have become [34]. There is a need to explore its feasibility and utility in non-UK contexts. Norway, as the other country where CRTs have been implemented nationally, is an obvious site for further testing of the scale.

While a fidelity review may in itself help services plan service improvement, there is also a pressing clinical need to develop and test resources to help CRT services meet the priorities of stakeholders, and achieve high model fidelity, with the aim of improving service outcomes. The development of a program manual, which can then be used in the training and supervision of programme staff, is advocated as a key tool in supporting high fidelity implementation of evidence based practices [43]. Development of a CRT manual is required, informed by the CORE CRT Fidelity Scale. The CORE CRT Service Improvement Programme Trial [30] will test a package of service improvement resources in a cluster randomised trial: 15 CRT teams will receive the package of resources over a 1-year period; 10 teams will act as controls. Fidelity reviews will be used to support and evaluate the service improvement intervention.

The development of the CRT Fidelity Scale also has two implications regarding research methods. First, concept mapping [29] proved to be a useful method of developing and defining the CRT service model. It allowed the views of several stakeholder groups to contribute to developing the model, and provided a transparent basis for making decisions about which of many competing elements of CRT services to prioritise for inclusion in a fidelity scale. Second, the development and piloting of the CRT fidelity scale provides a model for patient and public involvement in research and clinical audit, as recommended to improve the quality and credibility of research [44].

## Conclusion

Crisis Resolution Teams are a complex intervention, with evidence of effectiveness from trials [12], but for which implementation has been variable [21, 22], and which appear to have been less effective than hoped for when scaled up to national level [16, 18]. Implementation has been identified as the major barrier to translating scientific knowledge into patient benefit [2]. The CORE CRT Fidelity Scale can support implementation of CRTs by providing clear and detailed specification of the CRT model, a means to assess CRT services’ adherence to this model, and a means to support and focus CRT service improvement initiatives at the levels of practice and policy. It fills an unmet need, in the previous absence of a fidelity scale for CRTs, and can help in the overarching goal of improving support for people experiencing a mental health crisis.

## Additional files

Additional file 1: Table DS1. CORE CRT concept mapping: participant ratings – summary scores. (DOCX 21 kb)
Additional file 2: Table DS2. CORE CRT concept mapping: statements with significant between-group differences in importance ratings. (DOCX 16 kb)
Additional file 3: Table DS3. Changes to the CORE CRT Fidelity Scale post-piloting. (DOCX 16 kb)
Additional file 4: Table DS4. Scoring of fidelity items in the CORE CRT Fidelity Scale inter-rater reliability exercise. (DOCX 21 kb)
Additional file 5: Table DS5. Concept mapping participant prioritisation data: CORE Study data set. (XLSX 27 kb)
Additional file 6: Table DS6. Inter-rater reliability testing participant data: CORE Study data set. (XLSX 11 kb)

## Abbreviations

CRT: Crisis Resolution Team
ICC: intra-class correlation
NHS: National Health Service
