# Supplementary material for: Development of a measure of model fidelity for mental health Crisis Resolution Teams
Source: BMC Psychiatry. 2016 Dec 1;16:427. doi: 10.1186/s12888-016-1139-4 (PMC5133753; doi:10.1186/s12888-016-1139-4)
Supplement: Additional file 1: Table DS1. — CORE CRT concept mapping: participant ratings – summary scores. (DOCX 21 kb) [file 12888_2016_1139_MOESM1_ESM.docx]

**Table DS1: CORE CRT fidelity scale concept mapping statements: mean importance ratings for all participants (N=68) and participant groups**

| **Statement** | **Median rating*** | **Mean rating** | **S.D.** | **4-cluster grouping**** |
| --- | --- | --- | --- | --- |
| 1. The CRT responds quickly to new referrals | 5 | 4.63 | 0.83 | R |
| 2. CRT staff provide help for drug and alcohol problems | 2 | 2.04 | 1.26 | C |
| 3. The CRT has administrative staff dedicated to the CRT team | 2 | 2.34 | 1.30 | S |
| 4. The CRT has clear referral processes | 4 | 3.78 | 1.30 | R |
| 5. The CRT provides individualised care | 4 | 4.07 | 1.12 | C |
| 6. The CRT team has direct access to a pharmacist from whom the CRT staff and service users and carers can seek advice | 2 | 2.28 | 1.33 | S |
| 7. The CRT is easily accessible to all eligible referrers | 5 | 4.21 | 1.15 | R |
| 8. The CRT signposts CRT service users and their families to other sources of help | 3 | 2.84 | 1.11 | C |
| 9. The CRT has comprehensive risk assessment and risk management plans | 4 | 3.85 | 1.39 | S |
| 10. The CRT will consider working with any service user who would otherwise be admitted to adult acute psychiatric hospital | 4 | 3.87 | 1.31 | R |
| 11. The CRT helps service users access peer support | 1 | 1.97 | 1.27 | C |
| 12. The CRT has systems to ensure the safety of CRT staff members | 4 | 3.53 | 1.40 | S |
| 13. The CRT accepts direct referrals from service users and their families/carers | 3 | 3.13 | 1.68 | R |
| 14. The CRT helps plan service users’ and service responses to future crises | 3 | 3.35 | 1.18 | C |
| 15. The CRT assesses and helps ensure the safety and welfare of all children and vulnerable adults living with CRT service users | 4 | 3.49 | 1.34 | S |
| 16. The CRT accepts direct referrals from Primary Care services | 3 | 3.16 | 1.44 | R |
| 17. The CRT follows clear policies and procedures about confidentiality and information sharing | 3 | 3.09 | 1.30 | S |
| 18. The CRT has a clearly defined “gatekeeping” role to screen and make decisions about admissions to hospital, and assesses all patients before admission to acute inpatient psychiatric wards | 4 | 3.25 | 1.68 | R |
| 19.The CRT accepts direct referrals from organisations other than health services | 2 | 2.26 | 1.45 | R |
| 20. The CRT provides information to service users and carers regarding diagnosis and the nature of difficulties | 3 | 3.10 | 1.04 | C |
| 21. CRT staff attend all assessments for compulsory detention in person | 2 | 2.24 | 1.32 | R |
| 22. The CRT provides explanation and signposting to service users, carers and referrers for referrals which are not accepted | 3 | 2.87 | 1.08 | L |
| 23. The CRT promotes service users’ and carers’ understanding of medication and helps with problems or concerns about medication | 3 | 2.74 | 1.09 | C |
| 24. The CRT provides a telephone service 24 hours, 7 days a week | 5 | 3.94 | 1.38 | R |
| 25. The CRT conducts a comprehensive assessment for all service users accepted for CRT treatment | 4 | 3.78 | 1.36 | L |
| 26. The CRT reviews, prescribes and delivers medication for all service users when needed | 3 | 3.37 | 1.34 | C |
| 27. The CRT is open 24 hours, 7 days a week, to see service users in person and provide assessment and treatment | 5 | 3.84 | 1.50 | R |
| 28. Initial assessments by the CRT are carried out by qualified, experienced CRT staff | 5 | 4.25 | 1.06 | S |
| 29. The CRT offers support other than medication for all service users and will work with service users who decline medication | 4 | 3.72 | 0.97 | C |
| 30. The CRT responds promptly to current service users’ or carers’ requests for help from the service | 4 | 3.81 | 1.38 | R |
| 31. The CRT mostly assesses and treats service users in their own home | 3 | 3.13 | 1.42 | L |
| 32. The CRT offers emotional support to carers, and assesses carers needs, expectations and abilities to cope | 3 | 2.96 | 1.24 | C |
| 33. The CRT provides a thorough induction programme for new staff | 3 | 2.78 | 1.33 | S |
| 34. The CRT offers flexibility about the location and timing of assessments and treatment | 3 | 3.00 | 1.09 | L |
| 35. The CRT offers practical support for carers and directs them to other support services | 2 | 2.40 | 1.16 | C |
| 36. The CRT provides ongoing training and supervision in core competencies for CRT staff | 3 | 2.96 | 1.18 | S |
| 37. The CRT facilitates early discharge from hospital | 3 | 2.96 | 1.30 | R |
| 38. The CRT provides frequent visits to service users | 4 | 3.35 | 1.23 | L |
| 39. The roles, skills and experience of the CRT staff team cover key areas of mental health crisis care | 4 | 3.65 | 1.16 | S |
| 40. The CRT provides a same-day home visit for all service users discharged early from an acute ward with CRT support | 2 | 2.47 | 1.41 | L |
| 41. The CRT promotes service users’ choice regarding types of support and how visits and support are arranged | 3 | 3.12 | 1.13 | C |
| 42. The CRT has effective record keeping policies and procedures | 3 | 2.96 | 1.29 | S |
| 43. The CRT helps service users with social problems (including housing, finances, debt, welfare benefit claims, problems with employers) | 3 | 2.59 | 1.30 | C |
| 44. The CRT provides clear information about treatment plans and visits to service users and families | 4 | 3.51 | 0.97 | C |
| 45. The CRT is a distinct service which only provides crisis assessment and brief home treatment, until an immediate crisis is resolved, to people who would otherwise have been admitted to acute psychiatric hospital | 2 | 2.72 | 1.58 | R |
| 46. The CRT helps service users with everyday living tasks | 1 | 1.90 | 1.16 | C |
| 47. Visits by CRT staff allow time to discuss all service users’ or family concerns | 3 | 2.94 | 1.27 | C |
| 48. The CRT has procedures in place to help effective working with other acute mental health services | 3 | 2.99 | 1.20 | S |
| 49. CRT staff will accompany service users to important appointments | 1 | 1.60 | 0.90 | C |
| 50. The CRT promotes good therapeutic relationships between staff and service users and carers | 4 | 3.51 | 1.22 | C |
| 51. The CRT has procedures in place to help effective working with other community mental health services | 3 | 2.85 | 1.15 | S |
| 52. The CRT helps service users with vocational and social activities | 1 | 1.51 | 0.94 | C |
| 53. The CRT takes account of the local geographical context and the nature of its catchment area in service planning | 2 | 1.99 | 1.17 | S |
| 54. The CRT has procedures in place to help effective working with organisations other than mental health services | 2 | 2.13 | 1.06 | S |
| 55. The CRT provides extended visits to service users where necessary to ensure safety | 4 | 3.60 | 1.08 | C |
| 56. The CRT uses audits, reviews and research to inform service practice and strategic development | 2 | 2.03 | 1.21 | S |
| 57. The CRT can access a range of crisis services to help provide an alternative to hospital admission for service users experiencing mental health crisis | 4 | 3.48 | 1.28 | L |
| 58. The CRT closely involves and works with families and wider social networks in supporting service users | 4 | 3.35 | 1.34 | C |
| 59. The CRT has adequate staffing levels | 4 | 4.16 | 1.09 | S |
| 60. Service users and carers are involved in the development and management of the CRT service | 2 | 2.24 | 1.26 | S |
| 61. The CRT assesses and addresses service users’ physical health needs | 2 | 2.60 | 1.20 | C |
| 62. CRT communication systems promote teamwork and information sharing between CRT staff | 3 | 3.31 | 1.18 | S |
| 63. The CRT has systems to obtain and act on service users’ and carers’ feedback and complaints about the CRT service | 2 | 2.50 | 1.17 | S |
| 64. The CRT provides a range of psychological interventions | 3 | 2.66 | 1.27 | C |
| 65. The CRT is a multidisciplinary staff team | 3 | 3.44 | 1.30 | S |
| 66. The CRT plans aftercare for all service users | 3 | 3.00 | 1.30 | C |
| 67. The CRT supports service users in using a range of self-management programmes | 2 | 2.65 | 1.16 | C |
| 68. The CRT has a psychiatrist or psychiatrists in the CRT team, with adequate staffing levels | 5 | 4.29 | 1.04 | S |
| 69. CRT systems promote acceptable endings of CRT care for service users and carers | 3 | 3.18 | 1.11 | C |
| 70. The CRT takes account of equality and diversity in all aspects of service provision | 3 | 3.00 | 1.44 | S |
| 71. The CRT team employs people with personal experience of using mental health services, and carers of people who have used services (e.g. as peer support workers or recovery workers) | 2 | 2.16 | 1.36 | S |
| 72. The CRT has systems to promote consistency of staff and support provided to a service user during a period of CRT care | 3 | 2.91 | 1.38 | S |

* Concept mapping participants (N=68) were required to group statements (N=72) into five groups, each containing 14 or 15 statements, based on relative importance (5 = most important, to 1 = least important)

** The chosen 4-cluster concept mapping solution grouped statements into the following 4 clusters: R= Referrals and access; C = Content and delivery of care; S = Staffing and team organisation; L = Location and timing of help
