# Supplementary material for: Development of a measure of model fidelity for mental health Crisis Resolution Teams
Source: BMC Psychiatry. 2016 Dec 1;16:427. doi: 10.1186/s12888-016-1139-4 (PMC5133753; doi:10.1186/s12888-016-1139-4)
Supplement: Additional file 2: Table DS2. — CORE CRT concept mapping: statements with significant between-group differences in importance ratings. (DOCX 16 kb) [file 12888_2016_1139_MOESM2_ESM.docx]

**Table DS2: CORE CRT fidelity scale concept mapping: statements with significant differences* between broad participant groups’ importance ratings**

|  | **Mean importance ratings** | | | |  |
| --- | --- | --- | --- | --- | --- |
| **Statement** | **All participants (n=68)** | **Service users and carers (n=22)** | **Mental Health staff (n=30)** | **Other stakeholders (n=16)** | **How the statement is reflected in the fidelity scale** |
| 9. The CRT has comprehensive risk assessment and risk management plans | 3.85 | 2.64 | 4.57 | 4.06 | Included as a distinct fidelity item (#31) because rated moderately or very highly by all groups |
| 11. The CRT helps service users access peer support | 1.97 | 2.59 | 1.47 | 1.81 | Peer Support workers were included as one staff group scoring towards provision of a multidisciplinary staff team (item #29) but not given more weight because only scored moderately highly by one participant group |
| 12. The CRT has systems to ensure the safety of CRT staff members | 3.53 | 2.64 | 4.13 | 3.44 | Included as a distinct fidelity item (#32) because rated moderately or very highly by all groups |
| 13. The CRT accepts direct referrals from service users and their families/carers | 3.13 | 3.91 | 2.87 | 2.31 | Included as a criterion within item #3 because rated moderately highly by two participant groups |
| 15. The CRT assesses and helps ensure the safety and welfare of all children and vulnerable adults living with CRT service users | 3.49 | 2.95 | 3.87 | 3.37 | Completion of safeguarding assessments and staff training on safeguarding were included as criteria within item #31, because this statement was rated moderately highly by all groups |
| 17. The CRT follows clear policies and procedures about confidentiality and information sharing | 3.09 | 3.05 | 3.43 | 2.31 | Evidence of clear policies on confidentiality and information sharing were included as a criterion within item #14 because this was rated moderately highly by two participant groups |
| 24. The CRT provides a telephone service 24 hours, 7 days a week | 3.94 | 4.41 | 3.47 | 4.19 | Included as a distinct fidelity item (#5) because rated moderately or very highly by all groups |
| 28. Initial assessments by the CRT are carried out by qualified, experienced CRT staff | 4.25 | 4.68 | 4.17 | 3.81 | The quality of initial assessments was included as a distinct fidelity item (#11) because this item was rated highly by all groups |
| 30. The CRT responds promptly to current service users’ or carers’ requests for help from the service | 3.81 | 4.55 | 3.37 | 3.63 | Included as a distinct fidelity item (#9) because rated moderately or very highly by all groups |
| 37. The CRT facilitates early discharge from hospital | 2.96 | 2.23 | 3.30 | 3.50 | Rated moderately highly by two participant groups: included as a distinct fidelity item (#7) following further consultation on proposed fidelity items |
| 47. Visits by CRT staff allow time to discuss all service users’ or family concerns | 2.94 | 3.27 | 2.47 | 3.31 | Rated moderately highly by two participant groups: included as a distinct fidelity item (#21) following further consultation on proposed fidelity items |
| 56. The CRT uses audits, reviews and research to inform service practice and strategic development | 2.03 | 1.45 | 2.40 | 1.94 | Not included: rated as of moderately or very low importance by all groups |
| 58. The CRT closely involves and works with families and wider social networks in supporting service users | 3.35 | 2.77 | 3.47 | 3.88 | Included as a distinct fidelity item (#13) because rated moderately highly by all groups |
| 71. The CRT team employs people with personal experience of using mental health services, and carers of people who have used services (e.g. as peer support workers or recovery workers) | 2.16 | 2.73 | 1.67 | 2.25 | Peer Support workers were included as one staff group scoring towards provision of a multidisciplinary staff team (item #29) but not given more weight because only scored moderately highly by one participant group |
| 72. The CRT has systems to promote consistency of staff and support provided to a service user during a period of CRT care | 2.91 | 3.32 | 2.43 | 3.25 | Rated moderately highly by two participant groups: included as a distinct fidelity item (#36) following further consultation on proposed fidelity items |

* Differences between scores from 3 broad participant groups for each item were tested using Anova (significance level: p <0.05)
