# Supplementary material for: Development of a measure of model fidelity for mental health Crisis Resolution Teams
Source: BMC Psychiatry. 2016 Dec 1;16:427. doi: 10.1186/s12888-016-1139-4 (PMC5133753; doi:10.1186/s12888-016-1139-4)
Supplement: Additional file 3: Table DS3. — Changes to the CORE CRT Fidelity Scale post-piloting. (DOCX 16 kb) [file 12888_2016_1139_MOESM3_ESM.docx]

**Table DS3: Changes to the CORE CRT Fidelity Scale following piloting**

| **Fidelity item** | **New Criteria introduced** | **Changes to existing criteria or scoring guidance** |
| --- | --- | --- |
| 1. The CRT responds quickly to new referrals | New criterion added that CRTs must respond promptly to requests for assessment from emergency services, including willingness to meet people in public if in contact with the police, or ambulance crew.  (in response to new UK government guidance) | Requirement for a 1-hour response to high-risk referrals dropped; 4-hour response target for all referrals retained (in response to CRT managers’ feedback about feasibility) |
| 3. The CRT accepts referrals from all sources |  | Accepting referrals from emergency services is now included as a separate criterion, distinct from other non-health sources (in response to new UK government guidance) |
| 6. The CRT gatekeeps hospital admissions |  | CRT attendance at “s.136” Mental Health Act assessments now distinguished from attendance at other MHA assessments in two criteria (in response to feedback from stakeholders about specific importance of attending s.136 assessments) |
| 9. The CRT responds to requests for help from service users and carers whom the team is supporting |  | Requirement for the CRT to monitor response times to service users and families was dropped (in response to feedback from CRT managers and stakeholders that actual practice is more important than whether monitoring takes place). The criterion that service users and families report receiving a prompt response was retained. |
| 10. The CRT is a distinct service |  | One criterion about working with people who would otherwise be admitted to hospital is split into two criteria (with 50% and 90% thresholds) to give this more weight in scoring (in response to stakeholder feedback that CRTs often do not work sufficiently exclusively with the most acutely ill patients). |
| 12. The CRT provides clear information to service users and families | New criterion added that CRTs must provide service users with a specific time (or maximum 1-hour time window) for visits (in response to feedback from service users during pilot reviews that CRTs are often very imprecise in when they will arrive, resulting in long waits) | Requirement for CRTs to monitor missed and late visits was dropped ( to place more emphasis on actual practice than monitoring – in response to CRT manager and stakeholder feedback) |
| 13. The CRT closely involves families and social networks |  | Clarification in scoring guidance (criteria a and b) that CRTs should attempt to identify and make contact with family or other social contacts wherever possible, not just where involved people are immediately present (in response to stakeholder feedback about the importance of CRTs providing proactive social systems working) |
| 14. The CRT assesses carers’ needs |  | Two criteria regarding provision of a written support plan for carers were combined into one (to address observed floor effect from the pilot: these criteria were rarely met) |
| 15. The CRT reviews, prescribes and delivers medication |  | Minor revisions of scoring criteria to add clarity (in response to reviewers’ feedback from piloting) |
| 16. The CRT promotes service users’ understanding of illness and medication |  | Two changes were made in response to feedback and to address floor effects noted in the pilot; i) The requirement for CRTs to use structured side-effect assessments was dropped (in response to CRT managers and stakeholder feedback – to increase focus on whether any side-effect monitoring is done, not how); ii) The criterion on medication adherence split to two to aid clarity: a) is adherence documented; b) are strategies to promote adherence used where required |
| 17. The CRT provides psychological interventions |  | Thresholds and scoring guidance for criteria were amended to focus more on provision of psychological interventions to CRT service users rather than just whether or not there is a psychologist practitioner in the team. Teams are now credited for providing broadly defined psychological interventions, even if these are not provided by a psychologist. A psychologist in the team is still necessary to score 5 (in order to improve the discriminative ability of the item and in response to further specific stakeholder consultation) |
| 20. The CRT provides individualised care | A criterion was added to use the case note review to assess provision of individualised care (the criterion relying on service user and carer feedback was also retained) (in response to reviewers’ feedback about the value of case notes as an information source) |  |
| 22. The CRT prioritises good therapeutic relationships between staff and service users |  | Scoring guidance was adjusted to give additional weight to whether service users and carers report that staff are caring and professional (in response to stakeholder feedback) |
| 24. The CRT helps plan responses to future crises |  | The scoring criterion on relapse prevention plans was divided into two, with a lower threshold for completion of partial plans (in response to observed floor effects from piloting: few teams routinely help service users develop comprehensive relapse prevention plans) |
| 26. The CRT prioritises acceptability to service users about how CRT care will end |  | The criterion for CRTs to provide a service directory was changed to: all-source agreement that CRTs provide relevant information about other services (in response to CRT and stakeholder feedback that directories of services are unwieldy, soon out of date, and insufficiently individualised) |
| 31.The CRT has comprehensive risk assessment and risk management procedures |  | The scoring guidance was relaxed: fewer criteria were required to be met to score 3 or above (in response to observed floor effects in piloting, to improve the discriminative ability of the item) |
| 33. The CRT has effective record keeping procedures |  | The requirement for CRTs to have a written policy on record keeping was dropped (in response to feedback from CRT managers and stakeholders, to increase the emphasis on practice rather than protocols) |
| 34. The CRT works effectively with other community services |  | Scoring guidance was amended so that two perceived key criteria (A and E) must be met to score 4 or 5 on this item ( in response to stakeholder feedback about priorities for joint working with other services) |
| 35. The CRT takes account of equality and diversity | A criterion was added that service users and carers reported no experience of discrimination and that the CRT was sensitive to diversity (in response to further stakeholder consultation) |  |
