# Supplementary material for: Development of a measure of model fidelity for mental health Crisis Resolution Teams
Source: BMC Psychiatry. 2016 Dec 1;16:427. doi: 10.1186/s12888-016-1139-4 (PMC5133753; doi:10.1186/s12888-016-1139-4)
Supplement: Additional file 4: Table DS4. — Scoring of fidelity items in the CORE CRT Fidelity Scale inter-rater reliability exercise. (DOCX 21 kb) [file 12888_2016_1139_MOESM4_ESM.docx]

**Additional file DS4: Data from the CORE CRT Fidelity Scale inter-rater reliability testing**

**Table DS4.1 Scores of individual raters (n=16) in the CORE CRT fidelity scale inter-rater reliability vignettes**

| **Fidelity Item** | **V. Low (1)** | **Low (2)** | **Fair (3)** | **Good (4)** | **Excellent (5)** | **Mean score** | **Standard deviation** |
| --- | --- | --- | --- | --- | --- | --- | --- |
| 1 | 9 | 5 | 2 | 0 | 0 | 1.6 | 0.7 |
| 2 | 0 | 0 | 3 | 7 | 6 | 4.2 | 0.8 |
| 3 | 2 | 4 | 7 | 2 | 1 | 2.8 | 1.1 |
| 4 | 0 | 0 | 3 | 10 | 3 | 4.0 | 0.6 |
| 5 | 0 | 2 | 14 | 0 | 0 | 2.9 | 0.3 |
| 6 | 0 | 2 | 1 | 9 | 4 | 3.9 | 0.9 |
| 7 | 0 | 8 | 5 | 3 | 0 | 2.7 | 0.8 |
| 8 | 1 | 4 | 9 | 0 | 2 | 2.9 | 1.0 |
| 9 | 1 | 0 | 1 | 7 | 7 | 4.2 | 1.0 |
| 10 | 0 | 3 | 8 | 5 | 0 | 3.1 | 0.7 |
| 11 | 3 | 2 | 9 | 1 | 1 | 2.7 | 1.1 |
| 12 | 4 | 9 | 2 | 1 | 0 | 2.0 | 0.8 |
| 13 | 4 | 9 | 3 | 0 | 0 | 1.9 | 0.7 |
| 14 | 3 | 8 | 5 | 0 | 0 | 2.1 | 0.7 |
| 15 | 0 | 2 | 4 | 2 | 8 | 4.0 | 1.2 |
| 16 | 11 | 4 | 1 | 0 | 0 | 1.4 | 0.6 |
| 17 | 15 | 1 | 0 | 0 | 0 | 1.1 | 0.3 |
| 18 | 1 | 13 | 2 | 0 | 0 | 2.1 | 0.4 |
| 19 | 0 | 3 | 8 | 3 | 2 | 3.3 | 0.9 |
| 20 | 0 | 7 | 2 | 7 | 0 | 3.0 | 1.0 |
| 21 | 5 | 6 | 5 | 0 | 0 | 2.0 | 0.8 |
| 22 | 1 | 3 | 4 | 6 | 2 | 3.3 | 1.1 |
| 23 | 0 | 0 | 0 | 10 | 6 | 4.4 | 0.5 |
| 24 | 5 | 9 | 2 | 0 | 0 | 1.8 | 0.7 |
| 25 | 5 | 6 | 4 | 0 | 1 | 2.1 | 1.1 |
| 26 | 0 | 0 | 1 | 1 | 14 | 4.8 | 0.5 |
| 27 | 0 | 1 | 1 | 0 | 14 | 4.7 | 0.9 |
| 28 | 0 | 0 | 1 | 3 | 12 | 4.7 | 0.6 |
| 29 | 1 | 12 | 3 | 0 | 0 | 2.1 | 0.5 |
| 30 | 5 | 6 | 3 | 2 | 0 | 2.1 | 1.0 |
| 31 | 0 | 1 | 12 | 3 | 0 | 3.1 | 0.5 |
| 32 | 0 | 1 | 1 | 7 | 7 | 4.3 | 0.9 |
| 33 | 0 | 0 | 9 | 7 | 0 | 3.4 | 0.5 |
| 34 | 9 | 6 | 1 | 0 | 0 | 1.5 | 0.6 |
| 35 | 1 | 4 | 5 | 4 | 2 | 3.1 | 1.1 |
| 36 | 0 | 5 | 11 | 0 | 0 | 2.7 | 0.5 |
| 37 | 8 | 7 | 1 | 0 | 0 | 1.6 | 0.6 |
| 38 | 1 | 6 | 9 | 0 | 0 | 2.5 | 0.6 |
| 39 | 0 | 0 | 0 | 1 | 15 | 4.9 | 0.3 |

**Figure DS4.1 Mean score versus standard deviation for each item in the CORE CRT inter-rater reliability testing***

***** The points with a higher value of sd (Item) correspond to Items with higher variability.
